# Systems Toxicology of Male Reproductive Development: Profiling 774 Chemicals for Molecular Targets and Adverse Outcomes

**DOI:** 10.1289/ehp.1510385

**Published:** 2015-12-11

**Authors:** Maxwell C.K. Leung, Jimmy Phuong, Nancy C. Baker, Nisha S. Sipes, Gary R. Klinefelter, Matthew T. Martin, Keith W. McLaurin, R. Woodrow Setzer, Sally Perreault Darney, Richard S. Judson, Thomas B. Knudsen

**Affiliations:** 1 Oak Ridge Institute for Science and Education, Oak Ridge, Tennessee; 2 National Center for Computational Toxicology, U.S. Environmental Protection Agency (EPA), Research Triangle Park, North Carolina; 3 Lockheed Martin, Research Triangle Park, North Carolina; 4 National Health and Environmental Effects Research Laboratory, U.S. EPA, Research Triangle Park, North Carolina

## Abstract

**Background:**

Trends in male reproductive health have been reported for increased rates of testicular germ cell tumors, low semen quality, cryptorchidism, and hypospadias, which have been associated with prenatal environmental chemical exposure based on human and animal studies.

**Objective:**

In the present study we aimed to identify significant correlations between environmental chemicals, molecular targets, and adverse outcomes across a broad chemical landscape with emphasis on developmental toxicity of the male reproductive system.

**Methods:**

We used U.S. EPA’s animal study database (ToxRefDB) and a comprehensive literature analysis to identify 774 chemicals that have been evaluated for adverse effects on male reproductive parameters, and then used U.S. EPA’s in vitro high-throughput screening (HTS) database (ToxCastDB) to profile their bioactivity across approximately 800 molecular and cellular features.

**Results:**

A phenotypic hierarchy of testicular atrophy, sperm effects, tumors, and malformations, a composite resembling the human testicular dysgenesis syndrome (TDS) hypothesis, was observed in 281 chemicals. A subset of 54 chemicals with male developmental consequences had in vitro bioactivity on molecular targets that could be condensed into 156 gene annotations in a bipartite network.

**Conclusion:**

Computational modeling of available in vivo and in vitro data for chemicals that produce adverse effects on male reproductive end points revealed a phenotypic hierarchy across animal studies consistent with the human TDS hypothesis. We confirmed the known role of estrogen and androgen signaling pathways in rodent TDS, and importantly, broadened the list of molecular targets to include retinoic acid signaling, vascular remodeling proteins, G-protein coupled receptors (GPCRs), and cytochrome P450s.

**Citation:**

Leung MC, Phuong J, Baker NC, Sipes NS, Klinefelter GR, Martin MT, McLaurin KW, Setzer RW, Darney SP, Judson RS, Knudsen TB. 2016. Systems toxicology of male reproductive development: profiling 774 chemicals for molecular targets and adverse outcomes. Environ Health Perspect 124:1050–1061; http://dx.doi.org/10.1289/ehp.1510385

## Introduction

Exposure to chemicals during prenatal development may increase the risk of adverse outcomes, and biomonitoring studies suggest pregnant women are exposed to multiple environmental chemicals (Woodruff et al. 2011). Adverse trends in male developmental reproductive health have been reported for rates of testicular germ cell tumors (TGCT), low semen quality, and relatively common human developmental defects such as undescended testes (cryptorchidism) and malformations of the genital tubercle (e.g., hypospadias) (Sharpe and Skakkebaek 2008; Virtanen et al. 2005).

The testicular dysgenesis syndrome (TDS) hypothesis posits an interrelationship among these adverse outcomes, as manifestations of altered prenatal testicular development in humans (Aschim et al. 2004; Bay et al. 2006; Biggs et al. 2002; Kaleva and Toppari 2005; Scott et al. 2007; Sharpe and Skakkebaek 2008; Skakkebæk et al. 2001). Epidemiological studies, however, provide scant support for a shared mechanistic origin of the four elements contributing to the TDS hypothesis (decreased sperm counts/infertility/subfertility, and common developmental defects such as cryptorchidism and hypospadias, and increasing incidences of TGCT) (Akre and Richiardi 2009). On the other hand, human studies have reported associations between at least some adverse outcomes of male reproductive tract development: shortened anogenital distance (AGD) in boys with undescended testis (Jain and Singal 2013); reduced AGD/penile length in patients with hypospadias or cryptorchidism (Thankamony et al. 2014); and TGCT with cryptorchidism/hypospadias/genital malformations (Trabert et al. 2013). The TDS hypothesis is difficult to test experimentally because of the inaccessibility of the fetal testis during formation and organization (8–12 weeks gestation), the lengthy period between induction and manifestation of some adult outcomes (20–45 years), the lack of definitive etiology for prenatal studies underlying hypospadias/cryptorchidism, and the lack of animal models for TGCT (Sharpe and Skakkebaek 2008).

Evidence for reduced androgenicity in the human TDS hypothesis comes from linkage studies correlating TDS elements to familial mutations in the androgen receptor (AR) (Lottrup et al. 2013). Studies using animal models of prenatal endocrine dysfunction have shown increased incidence rates for cryptorchidism, hypospadias, and low sperm quality following exposure to environmental compounds that may be acting through an anti-androgenic mode of action (Gray et al. 2006; Hu et al. 2014; Mylchreest et al. 1998, 2000, 2002; Sharpe and Skakkebaek 2008; Virtanen et al. 2005; Wilson et al. 2008). For example, exposure of pregnant rats to dibutyl phthalate (DBP) during the critical period of male reproductive development resulted in reduced AGD, increased cryptorchidism and hypospadias, testicular atrophy with germ cell loss, and weight reductions in the epididymis, seminal vesicles, and prostate (Mylchreest et al. 1998, 2000). Exposure of pregnant rats to di(2-ethylhexyl) phthalate (DEHP) resulted in abnormal testes development with large aggregates or clusters of Leydig cells in the interstitial spaces, multinucleated germ cells in the seminiferous cords, and significant reductions in testosterone levels in the fetal rat testis (Parks et al. 2000). Phthalates may increase testicular testosterone production (low-dose effect) in fetal rats, but higher dosages decrease testosterone production in the fetal testis, thereby leading to reduced AGD and increased rates of cryptorchidism (Ge et al. 2007). The effect on fetal testosterone production has been linked to reduced expression of steriodogenic enzymes (CYP17, CYP11a, StAR) and insulin-like 3 (INSL3) (Wilson et al. 2008). INSL3, a Leydig cell hormone that drives development of the gubernaculum, may underlie phthalate-induced cryptorchidism (Johnson et al. 2010). These findings support the connectivity to prenatal disruption of androgen signaling (AR mutations in the human TDS hypothesis) or androgen homeostasis (phthalate syndrome in rats) during the critical period of sexual differentiation.

Further evaluation of the fetal rat testis proteome following prenatal exposure to DEHP revealed altered cellular differentiation and cell migration pathways, as well as decreased testosterone production consistent with an anti-androgenic effect (Veeramachaneni and Klinefelter 2014); however, those authors also found that plasma estradiol was elevated approximately 2-fold in rat fetuses exposed to DEHP (Veeramachaneni and Klinefelter 2014). Alterations in estrogen pathways may also contribute to the prenatal origins of at least some elements of TDS (humans) or phthalate syndrome (rats). Although the synthetic estrogen diethylstilbestrol (DES) can impair testosterone production in fetal rats, the estrogen receptor (ESR1) is not expressed in human fetal Leydig cells (Mitchell et al. 2013). As such, our understanding of male reproductive developmental toxicity following estrogenic and/or anti-androgenic exposures is incomplete (Ono and Harley 2013).

Abnormal testis development could have numerous other primary causes beyond prenatal disruption of AR or ESR1 pathways. A human genome-wide association study (GWAS) on a cohort of 488 patients with symptoms of TDS, comprising 212 patients with TGCT, 138 with cryptorchidism, 31 with hypospadias, and 107 with infertility found an association with genetic markers located in the region of *TGF*β*R3* (transforming growth factor, beta receptor III) and *BMP7* (bone morphogenetic protein 7) (Dalgaard et al. 2012). GWAS for developmental defects of the male reproductive system have also identified candidate genes for hormonal (*AR*, steroidogenesis, *ESR1*, and *ESR2*) and nonhormonal (*FGF8*, *FGFR2*, *BMP4*, and *BMP7*) pathways underlying hypospadias (Carmichael et al. 2013; Geller et al. 2014). The association of hypospadias to fibroblast growth factor (FGF) signaling pathways in humans is consistent with mouse studies in which various degrees of hypospadias have been observed in mutations that affect FGF signaling, either alone or in interaction with pathways directing the spatial patterning of male genital tubercle morphogenesis (e.g., SHH, BMP, WNT signals) (Kojima et al. 2010).

Male reproductive developmental disorders that involve elements in the hypothesized TDS (humans) or a phthalate syndrome (rats) thus reflect a complex disease process in which fundamental and varied genetic and environmental factors interact to perturb morphogenetic signals that spatially pattern the male reproductive tract, or otherwise disrupt endocrine signals that further regulate its growth and maturation during sexual diversification. Given this complexity, a systems analysis may help unravel the molecular and functional changes leading to male reproductive developmental toxicity (MRDT). The recent availability of high-throughput screening (HTS) data, quantitative structure-activity relationship (QSAR) models, and systems modeling tools enables mining of toxicity information across a vast chemical landscape (Collins et al. 2008; Kavlock et al. 2012; NRC 2007; Sturla et al. 2014).

In the present study we used an HTS-driven approach to unravel the potential connectivity of environmental exposures to diverse molecular pathways and cellular processes that are disrupted in male reproductive developmental toxicity. The aims of this study were to *a*) provide a comprehensive evaluation of structurally diverse chemicals with male reproductive developmental effects in laboratory animal studies; *b*) identify chemical–bioactivity and chemical–end point relationships that are anchored to altered developmental phenotypes of the male reproductive system; and *c*) provide a cohesive network of interactions underlying the molecular and functional changes predicted to invoke male reproductive developmental toxicity.

## Methods

### ToxRefDB Implementation

**Figure 1:**
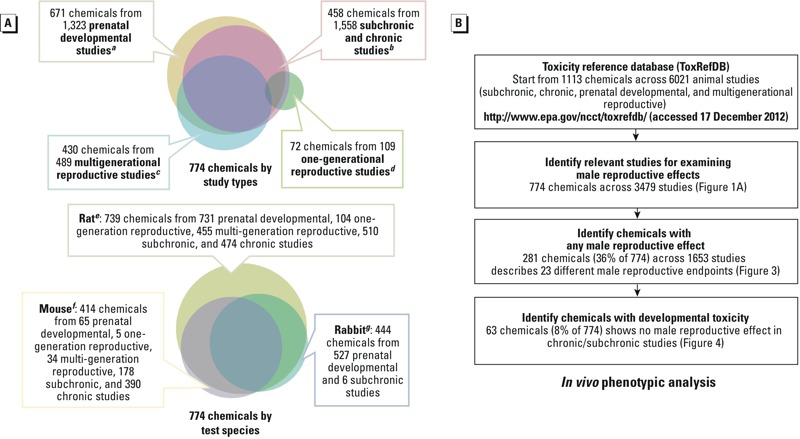
*In vivo* phenotypic analysis: chemical coverage and workflow. (*A*) Experimental protocols in general follow EPA Health Effects Test Guidelines OPPTS ***^a^***870.3700, ***^b^***870.3100, 870.4100, 870.4200, 870.4300, ***^c^***870.3800, and ***^d^***870.3550. Test strains include ***^e^***Alderly Park, CD, Fischer 344, Long-Evans, Sprague-Dawley, and Wistar for rats; ***^f^***B6C3F1, CD, and Swiss for mice; and ***^g^***Chinchilla, Dutch, Himalayan, and New Zealand White for rabbits. The prenatal developmental study type (OPPTS 870.3700 or equivalent) consisted mostly of rabbit (527) and rat studies (731), whereas both one-generational and multigenerational reproductive study types (OPPTS 870.3800, 870.3550, or equivalent) consisted primarily of rat studies (104 and 455, respectively). The ToxRefDB subchronic and chronic study types (OPPT 870.3100, 870.4100, 870.4200, 870.4300, or equivalent) consisted mostly of mouse (568) and rat studies (984). (*B*) Workflow for data-mining ToxRefDB.

All *in vivo* data for the current work was extracted from ToxRefDB (Toxicity Reference Database, http://actor.epa.gov/; Figure 1A,B). This collection of publicly available *in vivo* data built from thousands of guideline animal studies includes guideline chronic/cancer rat and mouse studies (Martin et al. 2009a), multigenerational reproduction rat studies (Martin et al. 2009b), and prenatal developmental studies (mostly pregnant rats and rabbits) (Knudsen et al. 2009). The ToxRefDB version used for the present study (release date 17 December 2012) had 6,021 animal studies on 1,113 chemicals, including 3,684 studies on pesticides and industrial chemicals, including perfluorinated compounds and phthalates; 151 studies with mammalian preclinical data for failed pharmaceutical agents; 434 studies from the National Toxicology Program (NTP); and 392 studies from open literature (PubMed). Figure 1A summarizes the ToxRefDB chemical coverage by study type and test species. Chemistry, biology, and usage features of these chemicals are accessible in the Aggregated Computational Toxicology Resource (http://actor.epa.gov/).

### Literature Search

**Figure 2:**
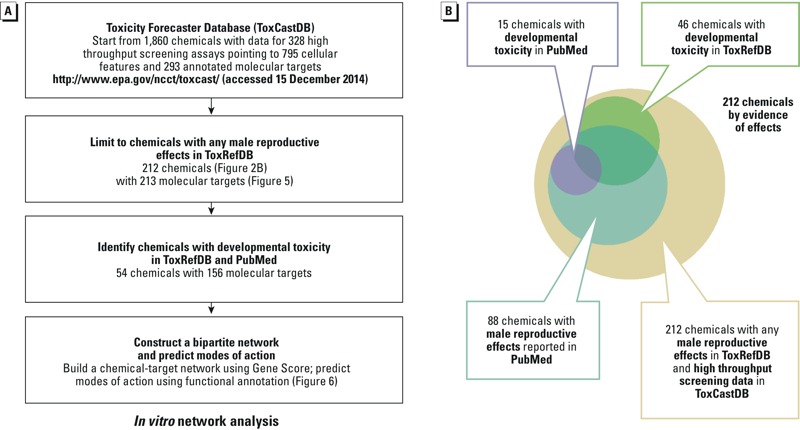
*In vitro *network analysis: workflow and evidence of effects. (*A*) Workflow for data-mining ToxCastDB. (*B*) A total of 2,099 articles were co-annotated with 88 of the 212 male reproductive toxicants in ToxRefDB (Figure 5), including 15 chemicals co-annotated with cryptorchidism, hypospadias, and urogenital abnormalities. The citations were listed in Excel File S1.

Because many ToxRefDB studies were not designed to assess male reproductive developmental end points, we searched the biomedical literature in PubMed for additional evidence of developmental toxicity for chemicals in ToxRefDB and ToxCastDB (Figure 2A,B; see also Excel File S1). Raw data were acquired from MEDLINE/PubMed Baseline Repository using MySQL on 15 May 2015 (http://mbr.nlm.nih.gov/) and processed in Microsoft Excel as Pivot Table. The chemicals were matched to the MeSH vocabulary to find the MeSH name and ID, either by name, by synonym, or CAS (Chemical Abstracts Service) number. Annotations of male reproductive end points were retrieved by searching on the MeSH categories “Urogenital Abnormalities,” “Spermatogenesis,” “Spermatozoa,” “Semen Analysis,” “Genitalia,” “Male,” and “Genital Neoplasms, Male.” Annotations of male reproductive developmental effects were retrieved by searching on the MESH terms “Cryptorchidism,” “Hypospadias,” and “Urogenital Abnormalities*.”* Because an end point could be co-annotated with a chemical when a lack of effect was discussed (i.e., studies with null findings or studies that did not assess any given effect), the annotation was manually curated to make sure whether or not the effect was found. To be inclusive, a chemical that showed an effect in one study would be counted as “positive” even if other comparable studies did not report that effect.

### Strategy to Define Male Reproductive Developmental Toxicity

The present study defined MRDT as all male reproductive birth defects reported in ToxRefDB and/or PubMed, as well as the sperm effects, histological effects, and tissue weight change reported exclusively in developmental studies in ToxRefDB. In other words, if an effect was reported in a developmental study and also reported in a nondevelopmental study, it would not be counted as MRDT. A total of 1,113 chemicals in ToxRefDB were filtered for *in vivo* phenotypic analysis based on the workflow described in Figure 1B. For chemicals that had a relevant study in ToxRefDB, the workflow selected those positive for any adverse male reproductive outcome. A chemical was considered positive for an effect when one or more study type in ToxRefDB recorded a relevant end point, regardless of dosage. That list was further parsed into a subset of chemicals where male reproductive developmental toxicity was evident in ToxRefDB for an early life stage exposure scenario. These data were culled from ToxRefDB studies of any type, but specifically for end points that could be linked to a prenatal developmental toxicity study or generational reproductive study. This strategy pointed to developmental defects including malformations, relative weight change, and histological effects for male reproductive organs (penis, testis, prostate, seminal vesicle), and any effects on sperm parameters or testicular cancers that were not strictly invoked in an adult-only chronic/cancer study type.

### Statistical Analysis for Male Reproductive End points

Comparisons for ToxRefDB studies across species (rat vs. mouse, rat vs. rabbit) and across generations (P, parental vs. F1, filial) used Pearson’s correlation in R-version 3.1.0 (R Core Team 2013). Co-occurrence of male reproductive end points was visualized in Microsoft Excel 2013 by grouping chemicals on a logarithmic scale (log_10_) by the least common end point. Chemicals passing through the workflow filter (Figure 1B) were subjected to two-directional hierarchical clustering in R version 3.1.0 using Euclidean distance for measure and Ward’s method for linkage analysis (Ward 1963). When the end point reflected a change to some parameter of male reproductive development in ToxRefDB, the lowest effect level (LEL) was determined for each category (cLELs for malformations, relative weight change, histological effects, and sperm parameters). If, for a particular chemical, the effects data were missing for a single category, the highest cLEL value for that chemical was multiplied by 10 to impute a value for clustering purposes. This does not imply that an actual effect is predicted, but rather enables the clustering across features in a hypothetical or virtual syndrome, where the missing value might be observed had a high enough dose been tested. Next, an “effect score” was calculated for each chemical and end point category to compute relative potency based on the cLEL values. The effect score was computed from the –log_10_ (cLEL mg/kg/day) dosage and normalizing the data set from 0 to 100 across the entire matrix. On this scale, 100 reflects the most potent chemical–end point category pair and 0 the least potent.

### ToxCastDB Implementation

All *in vitro* data for the current work was extracted from 1,860 chemical–bioactivity effects data in ToxCastDB (release date 15 December 2014) containing assay data and assay information (http://actor.epa.gov/dashboard/). The large battery of molecular and cellular HTS assays (Kavlock et al. 2012) has been used in earlier versions to develop predictive signatures of diverse chemical–bioactivity relationships in prenatal developmental toxicity (Sipes et al. 2011). The ToxCastDB version for the present study included 328 HTS assays pointing to 795 cellular features and 293 annotated molecular targets (BioAssay Ontology version 1.6). Some molecular targets among the 293 were assayed in multiple orthogonal platforms, whereas others may have been unique to one technology platform. The data processing pipeline (http://epa.gov/ncct/toxcast/data.html) included raw data quality assurance checks, normalization, automated curve fitting, visual plots of the concentration–response relationships, and calculation of concentration causing half maximal response in activity (AC_50_) or the lowest effect concentration (LEC).

### Gene Score Calculation

We used a panel of cytotoxicity assays in ToxCastDB to account for cytotoxicity-related assay interference. This accounted for nonspecific bioactivity that was presumably caused by integrated stress responses (Harding et al. 2003). The assay interference was assessed with a collection of 37 cellular features that indicated cytotoxicity, other forms of cell loss, or decrease in proliferation in several cell lines and primary cell types. For any given pairing of a chemical and a cytotoxicity end point, we defined a “hit” as a situation in which *a*) the best fit to the concentration–response data was given by either a Hill model or gain–loss model, and *b*) the maximum response predicted by the best-fitting model was greater than some critical threshold.

For a chemical *X* with two or more cytotoxicity hits, we analyzed the set of logarithms (log_10_) of the AC_50_ values corresponding to those hits. We let *m*(*X*) denote the median of this set and computed the median absolute deviation (MAD) of the set. Then, we computed *M*_global_ as the median of the MADs for *all* chemicals. Finally, for each chemical *X* and each cytotoxicity end point *A*, we computed a standardized score:


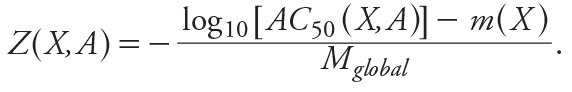
 [1]

For a chemical *X* with fewer than two cytotoxicity hits, we set *m*(*X*) = 3 for all *A*.

Because we considered multiple cellular features and many molecular targets, we computed “gene score” to summarize the ToxCastDB data. Gene score for chemical *X* and gene *g* was given by


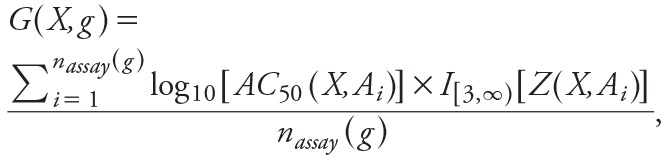
 [2]

where


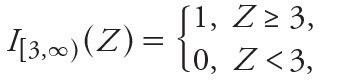


and *n*_assay_(*g*) was the number of cellular features corresponding to gene *g*, and {*A*_1_, …, *A_n_*__assay_(_ *_g_*_)_} was the set that contained all of cellular features. A hit with a large value of *G* occurred at concentrations significantly below cytotoxicity and therefore indicative of selective bioactivity. This excluded most of the cytotoxicity-driven hits, but allowed borderline cases to be included. The modified gene score as a micromolar concentration can be computed as

*G*´(*X*,*g*) = 10^^–log^_10_^[^^ *^^G^^* ^^(^^ *^^x^^* ^^,^^ *^^g^^* ^^)]^^. [3]

### Bipartite Network Visualization

The workflow for *in vitro* network analysis focused on an expanded list of ToxCast™ chemicals with male reproductive developmental effects identified from ToxRefDB (Figure 1B) and PubMed literature search to capture male developmental toxicants that might be missed or excluded from the chemical list (Figure 1B). Hierarchical clustering was performed on ToxCastDB effects using the same *R* metrics for the ToxRefDB analysis (Ward 1963; R Core Team 2013). A bipartite network for chemical–assay effects was then constructed using ForceAtlas layout in Gephi version 0.8.2 beta (Bastian et al. 2009). Functional annotation of the nodes (assay target) and edges (chemical target) in the network graph used the NIH/NIAID (National Institutes of Health/National Institute of Allergy and Infectious Diseases) Database for Annotation, Visualization and Integrated Discovery (DAVID) version 6.7 (Huang et al. 2009a, 2009b), selecting terms from significant (*p* < 0.05) functional annotation clusters generally based on pathways and biological processes.

## Results

### ToxRefDB Workflow for Male Reproductive Developmental Toxicity

A total of 774 ToxRefDB chemicals were identified in five study types that employed three test species (Figure 1A,B). ToxRefDB study types included prenatal developmental studies (maternal exposure during pregnancy; fetuses evaluated at term), one-generational studies (parental exposure, preconception through lactation; F1 filial generation evaluated postnatally); multigenerational reproductive studies (exposure continued to the F2 filial generation), subchronic studies (90-day adult exposure); and chronic studies (adult exposure up to 2 years). The relevant 3,479 studies came from testing on rats (2,274 studies), mice (672 studies), and rabbits (533 studies).

Twenty-three male reproductive end points, regardless of dosage, were reported for a total of 281 chemicals (36% of the 774) across 1,653 ToxRefDB studies. These 23 end points were classified into five categories for the male reproductive system, whether developmental or not: *a*) malformations, *b*) testicular tumors, *c*) sperm effects, *d*) histological effects, and *e*) relative weight change in male reproductive organs (Table 1; see also Excel File S2). Malformations encompassed reduced AGD, hypospadias, cryptorchidism, and abnormal nipple retention, all of which were linked to early life stage (prenatal, early postnatal) exposure scenarios. The testicular tumor types included adenoma, interstitial cell tumor, mesothelioma, and other (i.e., uncategorized in ToxRefDB). Although subchronic and chronic study types identified 31 chemicals that produced testicular tumors, seminomas (TGCT) were not reported in ToxRefDB studies. Therefore, the TGCT component of TDS could not be considered further. Sperm effects included alterations in morphology, motility, epididymal sperm count, testicular spermatid count, as well as semen sperm count. Histological effects were reported for the prostate, seminal vesicles, epididymis, and testes based on general features (e.g., inflammation, necrosis, and pigmentation) and spermatogenesis-specific abnormalities (e.g., aspermia, immature sperm, and retained spermatids). Tumor effects, sperm effects, and histological effects could result from early lifestage as well as adult exposure scenarios in ToxRefDB study designs; therefore, those adverse outcomes may indicate an adult manifestation of developmental toxicity and/or toxicity to the adult reproductive tract.

**Table 1:** ToxRefDB positive chemicals for male reproductive effects.

Male reproductive effect	Number of positive chemicals (mean lowest effect level; cLEL) (mg/kg/day)						
	Rabbit	Mouse	Rat	P1	F1	P1 or F1	MRDT
Malformation	1	0	14	NA	12	12	12
Hypospadias	0	0	2 (98)	NA	2 (98)	2	2
Nipple retention	0	0	3 (70)	NA	3 (70)	3	3
Decreased anogenital distance	0	0	9 (57)	NA	8 (232)	8	8
Cryptorchidism	1 (80)	0	7 (241)	NA	6 (285)	6	6
Testicular tumor	0	1	27	28	NA	28	NA
Mesothelioma	0	0	5 (6)	5 (6)	NA	5	NA
Adenoma	0	0	8 (25)	8 (25)	NA	8	NA
Interstitial cell tumor	0	1 (113)	15 (35)	16 (38)	NA	16	NA
Other	0	0	1 (11)	1 (11)	NA	1	NA
Sperm effect	1	15	42	34	24	51	36
Morphology	1 (3)	3 (39)	10 (64)	9 (67)	10 (42)	14	12
Motility	0	8 (102)	21 (44)	21 (56)	6 (31)	25	14
Testicular spermatid count	0	4 (40)	9 (84)	8 (111)	5 (58)	12	11
Epididymal sperm count	0	4 (79)	12 (64)	7 (29)	10 (159)	15	13
Semen sperm count	0	5 (58)	11 (90)	12 (65)	4 (36)	15	8
Histological effect	0	34	95	103	27	111	20
Prostate	0	2 (398)	17 (30)	15 (44)	6 (31)	19	7
Seminal vesicle	0	3 (158)	8 (25)	10 (41)	1 (50)	11	2
Epididymis	0	6 (153)	25 (20)	25 (23)	9 (17)	27	7
Epididymis (spermatogenesis-specific)	0	5 (62)	22 (67)	22 (68)	4 (50)	26	8
Testis	0	11 (159)	29 (56)	35 (69)	4 (127)	39	8
Testis (spermatogenesis-specific)	0	23 (96)	65 (56)	71 (57)	17 (32)	77	16
Relative weight change in tissue	0	42	176	174	58	196	50
Prostate	0	3 (342)	40 (64)	29 (100)	16 (42)	42	16
Seminal vesicle	0	4 (274)	36 (41)	29 (36)	14 (138)	39	20
Epididymis	0	5 (294)	34 (54)	25 (45)	21 (81)	37	27
Testis	0	36 (88)	153 (56)	154 (56)	47 (83)	172	36
Total	2	64	224	221	84	248	63
NA, not applicable. Includes 248 of the 281 chemicals positive for male reproductive effects with dosage information for prenatal and generational (P1, F1) studies (F2 and F3 data not shown); mean cLELs are listed in parentheses. A chemical was found to produce a male reproductive effect if produced any of the listed search terms in ToxRefDB in Excel File S2, regardless of dosage. MRDT (male reproductive developmental toxicity): sperm effects, histological effects, and tissue weight change were reported exclusively in developmental studies, plus all malformations; tumor location and methods used for sperm count were not specified in ToxRefDB; general histological effects include inflammation, necrosis, and pigmentation, in contrast with spermatogenesis-specific histological effects such as aspermia, immature sperm, and retained spermatids.							

Table 1 categorizes 248 (88%) of the 281 chemicals that are both positive for male reproductive effects and have dosage information for prenatal and generational (P1, F1) studies. All 23 male reproductive effects registered by the ToxRefDB chemicals were reported in the rat as a test species. Most effects were also reported in mouse; however, no malformation and only one of the four tumor types were reported in the mouse. Only 41 (18%) of the 224 male reproductive toxicants in rats are also male reproductive toxicants in mice. These 41 chemicals accounted for 64% of the 64 male reproductive toxicants in mice. Despite this discordance for the rat, the total number of chemicals associated with sperm effects, histological effects, and relative weight change correlated strongly between these rodent species (Pearson’s *R* = 0.93 across 15 effects). The mean cLEL was generally lower for the rat (50 ± 1 mg/kg/day) versus the mouse (120 ± 1 mg/kg/day), indicating that the rat was the more sensitive species. Rabbit prenatal studies reported male reproductive effects for two chemicals only (spiroxamine and sulfluramid) and could not be directly compared. Testicular tumors and malformations were specific to evaluation of either the parental (P1) or filial (F1) generation, respectively, in rat studies (mouse not tested). The number of chemicals associated with sperm effects, histological effects, and relative weight change correlated strongly between the P1 and F1 generations (*R* = 0.89). Mean cLELs for these end points was also similar (53 ± 1 mg/kg/day and 54 ± 1 mg/kg/day, respectively; Table 1).

**Figure 3:**
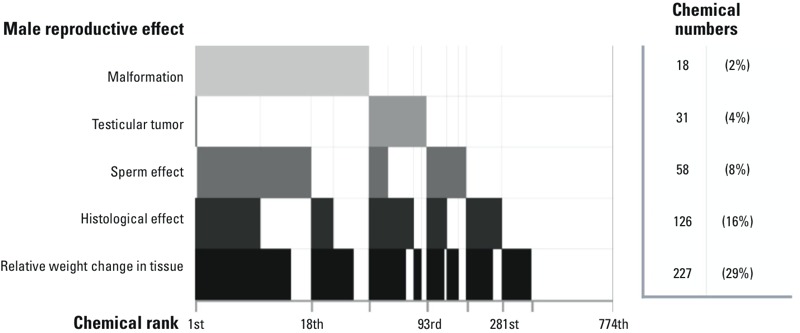
Co-occurrence of male reproductive effects in ToxRefDB. A chemical was found to produce a male reproductive effect if it was associated with any of the listed search terms in Excel File S2 in ToxRefDB, regardless of dosage. Chemicals may be associated with multiple effects. Chemicals were ranked in the order of association with the least common effect (i.e., malformations), followed by the next (i.e., testicular tumors), and so on.

The similarities between the human TDS hypothesis and the rodent phthalate syndrome motivated an examination of the co-occurrence of male reproductive effects across the 281 chemical list (Figure 3). A broken hierarchy was evident, with relative weight change accounting for the broadest response (227 chemicals, or 81% of 281). This was followed in turn by histological effects (126 chemicals, or 45%), sperm effects (58 chemicals, or 21%), testicular tumors (31 chemicals, or 11%), and malformations (18 chemicals, or 6.4%). Only vinclozolin produced both malformations and testicular tumors; and only 6 chemicals produced both malformations and sperm effects (Figure 3). The hierarchy of male reproductive effects resembled what might be expected in the human TDS hypothesis (i.e., testicular atrophy > sperm effects > tumors > malformations) (Akre and Richiardi 2009), with the caveat that seminomas (TGCT) could not be evaluated.

**Figure 4:**
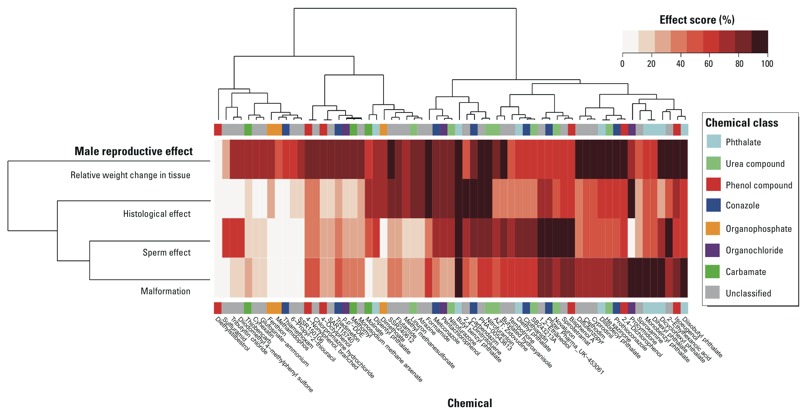
Phenotypic hierarchy of 63 male reproductive developmental toxicants in ToxRefDB. An unsupervised two-directional heat map was constructed based on hierarchical clustering of 63 chemicals by their effect scores for malformations, sperm effects, histological effects, and relative weight change in male reproductive tissues, using Euclidean distance for measure and Ward’s method for linkage analysis.

Figure 4 illustrates the hierarchical data structure of 63 MRDT chemicals by effect score in a heat map annotated by chemical class and adverse outcome. This analysis excluded 218 out of the 281 chemicals where adult male reproductive toxicity was observed in a chronic/cancer or subchronic study. This resulted in a subset of 63 MRDT chemicals that included 8 of 13 phthalates from the original (774 chemical) data set. The arrangement by effect score was similar to the broken hierarchy for the 281 male reproductive toxicants (Figure 3). Butylbenzyl phthalate stood out among the 63 chemicals in general, and the 13 phthalates in particular, as the sole MRDT with a high effect score across all four phenotypic categories (center of heat map in Figure 4). An enriched “phthalate cluster” (right side of the heat map in Figure 4) included diisobutyl phthalate, dicyclohexyl phthalate, dihexyl phthalate, and monobenzyl phthalate driven by a high effect score for malformations (primarily) and relative organ weight change (secondarily). In contrast to butylbenzyl phthalate, DES was the sole MRDT with a low effect score across all four phenotypic categories (left side of heat map in Figure 4). It was distinct from other phenolic compounds with estrogenic activity (4-nonylphenol, 4-octylphenol, bisphenol A, and isoeugenol) (Laws et al. 2000), which showed a high effect score in at least one phenotypic category. The phenolic estrogens 4-nonylphenol and 4-octylphenol co-clustered strongly with SAR115740 (TRPV1 antagonist) and chlorpromazine hydrochloride (dopamine antagonist), respectively.

### ToxCastDB Bioactivity Profile of Male Reproductive Toxicants

**Figure 5:**
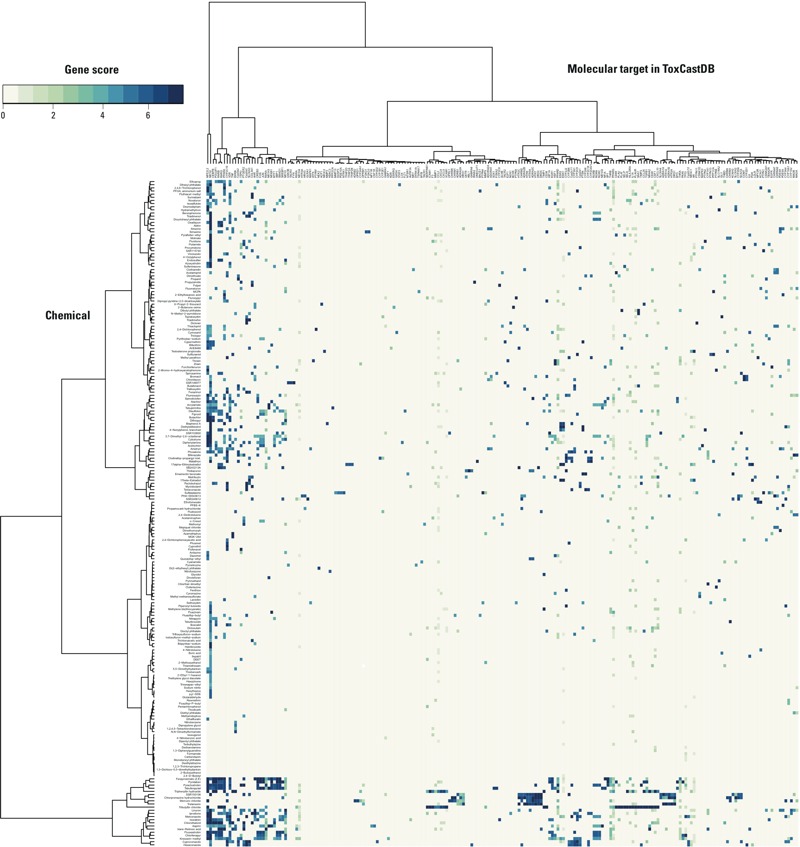
Unsupervised two-directional heatmap from hierarchical clustering of 212 male reproductive toxicants in ToxCastDB. Data for 212 male reproductive toxicants in ToxCastDB were used to construct a heat map to visualize the complex chemical–effects relationships on 213 molecular targets. The heat map was generated with the “gplot” package in R version 3.1.0, using Euclidean distance for measure and Ward’s method for linkage analysis. The intensity of blue color indicated the value of gene score.

To visualize the ToxCastDB bioactivity profiles on the 281 male reproductive toxicants, we performed hierarchical clustering of the chemicals based on gene score (Figure 5). ToxCast HTS data were available for 212 (75%) of the 281 toxicants (Figure 2B). Strong bioactivity was detected across a broad set of assays, most of which were co-culture cell–cell signaling platforms (BioSeek, BSK), multiplex transcriptional reporter platforms (Attagene, ATG), and HepG2 cell stress assays at 24 hr or 72 hr (APR and Tox21). The heatmap shown in Figure 5 is a hierarchical cluster of the 212 chemicals by 213 molecular targets based on gene score. The following are notable features in the heat map:

NFE2L2 and NR1I2: nuclear receptors in xenobiotic metabolism and response to oxidative stress
CREB3, FOS, and JUN: FOS/Jun (AP-1 transcription factor) family members that crosstalk with CREB
ESR1 and ESR2: estrogen receptors
CYP3A1, CYP2J2, CYP2B6, CYP2C11, and CYP2A1: cytochrome-P450s
SERPINE1, ICAM1, F3, MMP3, CCL2, CXCL5, CXCL9, and VCAM1; and CD69, PLAU, PLAT, SELP, SELE, HLA-DRA, IL1A, PLAUR, SAA1, TIMP2, CCL26, TGFB1, NR4A2, CSF1, and CXCL10: tissue remodeling during inflammation and angiogenesis
RXRA and NR1H4: retinoid X receptor and a binding partner; RORB, RORA, and RORC: retinoic acid receptor-related orphan receptors; RARB, RARG, and VDR: retinoic acid and vitamin D receptors; and CEBPB, POU2F1, and PPARA
HTR2C, CHRM2, ADRA2B, HTR6, HTR5A, ADRA2C, HTR7, DRD2, and DRD4; SLC6A4, ADRA1A, HTR1A, ADRA1B, HTR2A, and DRD1; OPRL1, CHRM5, CHRM1, ADRB1, ADRA2A, and HRH1; and CHRM4, CHRM3, KCNH2, SLC6A4, CYP2D6, SCN1A: four clusters of neuronal GPCRs (G-protein-coupled receptors: adrenergic, cholinergic, histamine, and serotonin receptors).

In general, the promiscuity of chemical–target hits was greater on the chemical side than the molecular target side. A subset of chemicals in the NFE2L2 and NR1I2 cluster could also be found in the RAR, RXR, and AP-1 clusters. Many neuronal GPCR chemical actives were found in the HTR2C cluster, followed by the SLC6A4, OPRL1, and CHRM4 clusters. Some clusters and molecular targets responded to specific chemicals, such as the SERPINE1 cluster (tributyltin chloride and triphenyltin hydroxide), FOX01 and FOX03 (simazine), PTPN1 and PTPN4 (sulfasalazine), and TFAP2B and TFAP2D (17 alpha-ethinylestradiol). Thiram and ziram produced similar gene scores over six molecular targets. We did not observe an evident clustering pattern for MRDT versus male reproductive toxicants (Figure 5).

### Literature Search for Male Reproductive Developmental Toxicants

Of the 212 male reproductive toxicants in ToxRefDB with ToxCastDB data (Figure 2B), we found 88 annotated by MeSH terms describing male reproductive effects in 2,099 articles in PubMed (see Excel File S1). We filtered this list by selecting chemicals with developmental toxicity and found 15 chemicals that were co-annotated with the MeSH terms “Cryptorchidism,” “Hypospadias,” and “Urogenital Abnormalities.” Eight of these chemicals were not previously identified as MRDT chemicals in ToxRefDB, thus increasing the total number of MRDT chemicals from 63 to 71 (of which 54 had ToxCast™ data).

### Human Homologs of ToxCast™ Assay Molecular Targets

**Figure 6:**
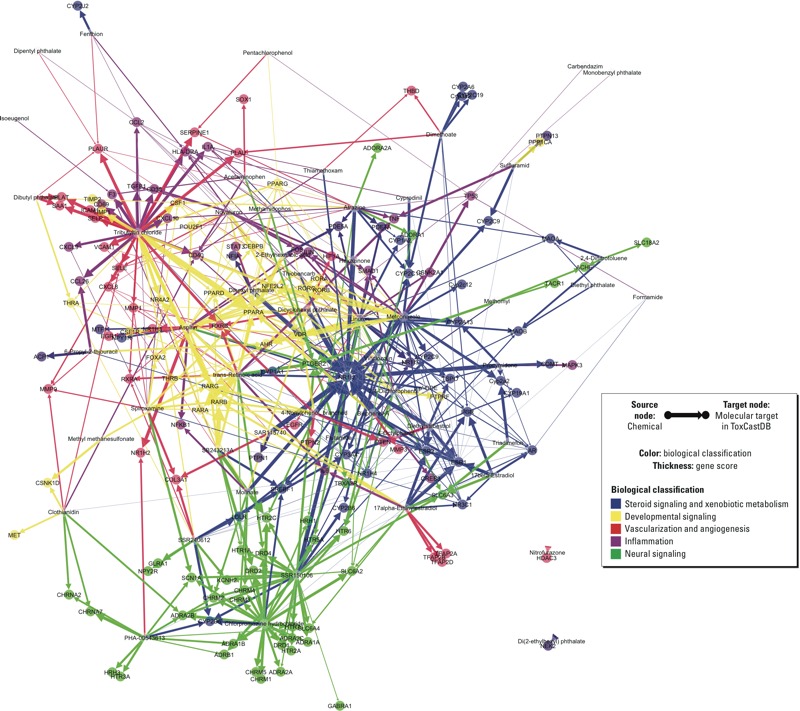
Quantitative bipartite network of 54 male reproductive developmental toxicants and 156 molecular targets in ToxCastDB. A bipartite network consisting of 54 male reproductive developmental toxicants (source nodes) and 156 molecular targets (target nodes; bold) was constructed with ForceAtlas layout using Gephi version 0.8.2 beta, with 5 predicted modes of action in different colors. The thickness of each chemical-to-molecular target arrow was proportional to the corresponding gene score.

Fifty-four MRDT chemicals from ToxRefDB and PubMed were mapped to 156 molecular targets in ToxCastDB (Figure 6). A list of the official gene symbols for these molecular targets was queried in the DAVID website (http://david.abcc.ncifcrf.gov/). Functional annotation by *Homo sapiens* captured 149 of the 156 gene symbols. Of the 7 IDs not captured or unmapped: *a*) *CXCL8*, formerly interleukin-8 (*IL-8*), was renamed by the Chemokine Nomenclature Subcommittee of the International Union of Immunological Societies, and the approved HUGO gene symbol is *CXCL8*; it functions as a major mediator of the inflammatory response and potent pro-angiogenic factor; *b*) *CYP2C6V1* is an allelic variant of *CYP2C19*, which is associated with drug (anticonvulsant) metabolism; *c*) Cyp2a1 is the rat homolog of human CYP2A13 and functions in the steroid/lipid/cholesterol/retinol metabolic pathways; *d*) rat Cyp2a2 is a sexually dimorphic CYP (male-specific liver expression starting at puberty) that has steroid hydroxylase activity (e.g., testosterone 15-alpha-hydroxylase); homologs are Cyp2a4 (mouse) and CYP2A6, CYP2A7, and CYP2A13 (human); *e*) rat Cyp2c11 is a male-specific steroid (testosterone) alpha-hydroxylase homologous to CYP2C9 (human), which converts testosterone to 2-alpha-hydroxyl and 16-alpha-hydroxyl metabolites; *f*) rat Cyp2c12 is a female-predominant liver P450 that is active in the 15-beta-hydroxylation of steroid sulfates (prominent in rat preovulatory ovarian follicles), but no information is available on a human homolog; and *g*) Cyp3a2 is another male-specific steroid (testosterone) alpha-hydroxylase in rats, and in humans the CYP3A cluster consists of four genes (*CYP3A43*, *CYP3A4*, *CYP3A7*, and *CYP3A5*); these enzymes hydroxylate testosterone to less active metabolites.

### Bipartite Network of Male Reproductive Developmental Toxicity

A bipartite network of the 54 MRDT chemicals and the 156 molecular targets in ToxCastDB was constructed in Gephi (Figure 6). In this network visualization of the target heat map (Figure 5), global inferences are extended to local relationships between different groups of molecular targets. Edge thickness of each chemical–target vector is proportional to the corresponding gene score, and the spatial distribution of nodes is based on connectivity. Nodes positioned centrally in the network represented the more commonly hit molecular targets: PPARs (peroxisome proliferator-activated receptors), RARs (retinoic acid receptors), RXRs (retinoid X receptors) in developmental regulation (yellow domain in Figure 6); as well as many nuclear receptors and crytochrome P450s in steroid homeostasis and xenobiotic metabolism (blue domain in Figure 6), such as NR1I2, ERs, AR, CYP19A1, and CYP3A5. Nodes positioned near the network’s rim had limited connectivity, implying greater specificity: GPCRs in neural activity (green domain in Figure 6), inflammation and angiogenesis (purple and red domains).

## Discussion

Mining ToxRefDB revealed a phenotypic hierarchy of male reproductive developmental disorders. The pleiotropic *in vitro* bioactivity profiles suggested that complex arrays of molecular targets contributed to adverse outcomes of the developing male reproductive system in rodents. Estrogenic and anti-androgenic activities, although extensively studied in male reproductive toxicology, were only a fraction of the potential molecular targets found (e.g., nuclear receptors, GPCRs, vascular remodeling proteins, and cytochrome P450s). We conclude from this analysis that phenotypic features of a human TDS-like hierarchy inferred from the pattern of MRDT evident in ToxRefDB animal studies connect with multiple pathways in addition to estrogen and androgen, leading to adverse male developmental outcomes. This highlights a need for additional research, including use of computational systems–based models, to better characterize the mechanistic causes of male reproductive developmental disorders.

Different modes of action have been ascribed to various aspects of MRDT (Albert and Jégou 2014; Gray et al. 2006; Kapp 2010; Kongsbak et al. 2014; Wilson et al. 2008). The present study confirmed the connections in rodents to ESR1 and ESR2, and to a lesser degree AR. The inferred role of ER signaling is consistent with recent findings that estrogen synthesis is a key target of prenatal exposure to phthalates in the fetal rat testis proteome (Klinefelter et al. 2012; Veeramachaneni and Klinefelter 2014). Steroid hormone metabolism involves multiple nuclear receptors and cytochrome P450s, many of which were found to be molecular targets of MRDT chemicals. CYP1A1, CYP19A1, CYP3A4, and COMT are involved in the synthetic pathway of estrogen and testosterone; NR1I2 (PXR, pregnane X receptor) and NR1I3 (CAR, constitutive androstane receptor) regulate the transcription of cytochrome P450s, such as CYP3A4, CYP2Cs, and CYP2B6 (Wang and LeCluyse 2003; Willson and Kliewer 2002). NR1I2, NR1I3, as well as PPARs are all involved in fatty acid and steroid metabolism (Handschin and Meyer 2005). Although NR1I2 and NR1I3 are xenobiotic receptors investigated in cancer, drug interaction, obesity, and metabolic diseases (Chai et al. 2013; Gao and Xie 2012), in the present study we infer a potential connectivity with many of the MRDT chemicals.

Our results support a developmental connection to male reproductive developmental disorders in rodents beyond AR and ER (Cohn 2011; Kilcoyne et al. 2014; Kojima et al. 2010; van den Driesche et al. 2015). Retinoic acid signaling is involved in morphogenesis, growth, differentiation, and tissue homeostasis, which include genital tubercle development and different phases of spermatogenesis (Liu et al. 2012; Mark et al. 2006, 2015). RXRs are binding partners of PPARs, RARs, thyroid hormone receptor, and vitamin D receptor (Abbott 2009), all of which were located at the central region of the bipartite network. AHR (aryl hydrocarbon receptor), PPARG, and CEBPB are all highly expressed in the placenta, which represents a potential MRDT target given its role as an endocrine organ (Scott et al. 2009; Storvik et al. 2014). Crosstalk between the AHR and ESR1/ESR2 signaling pathways (Gräns et al. 2010; Swedenborg et al. 2012) requires further investigation. Although AHR, ESR1, and ESR2 are promiscuous targets in the bipartite network, the measured AHR downstream targets (CYP1A1) and Per-Arnt-Sim (PAS) domain-containing proteins (HIF1A and KCNH2) show limited connectivity with one another, consistent with the recent molecular genetic screening studies (Geller et al. 2014; Madak-Erdogan et al. 2013). Further HTS experiments are needed to expand coverage for the TDS-related candidate genes, such as *SHH*, *FGF10*, *BMP7*, *HOXA4*, *DGKK*, and *TGFBR3* (Carmichael et al. 2013; Dalgaard et al. 2012; Geller et al. 2014).

Vascularization plays an important role in the early stage of testis and placenta development (Brennan et al. 2002; McClelland et al. 2012; Reynolds and Redmer 2001). Although the testis and placenta play different roles in prenatal regulation of steroidogenesis in humans and rodents (Scott et al. 2009), the present study supports the hypothesis that vascular disruption is a key event in MRDT, similar to pregnancy loss and other birth defects (Abe et al. 2013; Kleinstreuer et al. 2011; Morita et al. 2014). Several molecular targets were identified in the IL-6 pathway (CSNK2A1, MAPK3, IL6, JUN, STAT3, and FOS), but previous knockout studies failed to show that the IL-6 pathway was critical to rodent embryo development (Sakurai et al. 2012, 2013; Yoshida et al. 2011). In comparison, the finding of molecular targets in the MAPK pathway (MAPK3, IL1A, JUN, NFKB1, TGFB1, TNF, TP53, and FOS) is consistent with previous toxicity studies in Sertoli cells (Qi et al. 2014) and embryo limb bud cells (Liu and Kapron 2010). Pathways underlying developmental angiogenesis and inflammation have crosstalk with one another as well as other biological processes regulated by growth factors, GPCRs, and nuclear receptors (Knudsen and Kleinstreuer 2011). For example, PPARs play a regulatory role in angiogenesis (Bishop-Bailey 2011), and NR1H4 (FXR, farnesoid X receptor) down-regulates genes involved in inflammation (Hollman et al. 2012). These observations indicate multiple molecular initiating events and intermediate key events leading to MRDT adverse outcomes, supporting the use of the adverse outcome pathways (AOPs) approach (Ankley et al. 2010; Sturla et al. 2014) as a model framework to assess potential MRDT chemicals.

Dopamine, serotonin, and gonadotropin-releasing hormone receptors have been studied in the context of “neuroendocrine disruption” (Waye and Trudeau 2011). GPCR signaling is also involved in estradiol regulation of steroidogenesis (Vaucher et al. 2014). In the present study we identified several neuronal GPCRs targeted by a small number of MRDT chemicals in rodents, clustering at the lower rim of the bipartite network. Many of these chemicals are pharmaceutical agents that display strong neural or inflammatory activity, such as chlorpromazine hydrochloride (dopamine antagonist), SSR150106 (chemokine receptor antagonist), and SSR240612 (bradykinin B1 receptor antagonist). The molecular targets of neural activity were located apart from other predicted modes of action in the network graph, suggesting a unique bioactivity profile for a separate class of MRDT chemicals.

Unraveling the multiple pathways and processes underlying adverse prenatal developmental outcomes predicted from ToxCastDB–ToxRefDB signatures is a multi-scale problem (Sipes et al. 2011). The concept of bipartite networks (Fuxman Bass et al. 2013) has been used in the functional annotation of protein interaction networks (Kelley and Ideker 2005; Sharan et al. 2007) and in statistical modeling of complex social networks (Easley and Kleinberg 2010; Latapy et al. 2008). We applied the network concept to quantitatively visualize one-way interactions between chemical(s) and molecular target(s) in the ToxCastDB HTS data set and revealed novel interactions between different clusters of molecular targets across chemical classes, pathways, target score, and connectivity. This concept can also be applied for interpreting other high-dimensional data sets in computational toxicology.

The pattern of male reproductive effects in ToxRefDB varies by developmental stages and by test species, which is consistent with the differences in male reproductive development between rats, mice, rabbits, and humans. For instance, both mice and rats exposed to DBP exhibit male reproductive tract defects, including enlarged seminiferous cords and multinucleated germ cell formation, but fetal testis steroidogenesis is inhibited only in rats (Johnson et al. 2012). The human fetal testis is also susceptible to the seminiferous cord and germ cell effects of DBP, but it is relatively insensitive to the anti-steroidogenic effect (Spade et al. 2014; van den Driesche et al. 2015). The placenta plays an important role in regulating fetal testis steroidogenesis in humans, but not in rodents (Scott et al. 2009). Although rabbits have been reported as a sensitive model for many male reproductive effects (Foote and Carney 2000; Veeramachaneni 2008), almost all rabbit studies in ToxRefDB belong to the prenatal developmental study type and thus were not designed to capture male reproductive end points, particularly sperm effects. Therefore, it is not surprising that ToxRefDB records only two male reproductive toxicants in rabbits.

According to the TDS hypothesis, TDS pathogenesis begins during fetal development, involving symptoms of both newborn (cryptorchidism, hypospadias) and young adult males (impaired spermatogenesis, TGCT) (Akre and Richiardi 2009; Sharpe and Skakkebaek 2008). Thus, animal studies may miss the TDS-related effects of a chemical if they are not specifically designed to capture an extended scenario of early-life exposure and later-life effects, such as TGCT (a failure of some gonocytes to differentiate during postnatal/pubertal development). In contrast, testicular adenoma, interstitial cell tumor, and mesothelioma can all be detected in ToxRefDB (Table 1). These tumors originate from somatic cell mutations, potentially occurring at later life stages (Clegg et al. 1997; Meeks et al. 2012). On the other hand, the aggregate distribution of malformations, sperm effects, and histological effects were better correlated to the TDS hypothesis. Taken together, an extended one-generation reproductive toxicity study (OECD 2011) may be a suitable tool for validating present HTS findings for MRDT (Mitchard et al. 2012; Spielmann 2009). Sperm effects strongly correlate with malformations and histological effects, both of which are important indicators of early-stage testicular toxicity. Sensitivity of the rat as a species in detecting sperm effects and histological effects was stronger than the mouse based on number of positive chemicals and mean cLELs. With the caveat that poor semen quality assessed in adult men cannot be attributed to early-life perturbation in the absence of sufficient maternal exposure history, this supports the case for using sperm effects as a noninvasive assessment to improve surveillance of human reproductive function at a population level (CDC 2014).

In conclusion, ToxRefDB and ToxCastDB provide a novel resource for exploring phenotypic linkages of MRDT to potential mechanistic pathways. This study reveals a phenotypic hierarchy of male reproductive effects across animal studies that were consistent with the human TDS hypothesis. Although a number of recent studies have demonstrated that both hormonal and nonhormonal signaling is involved in male reproductive developmental defects (Carmichael et al. 2013; Dalgaard et al. 2012; Geller et al. 2014), the present study extends these findings, showing androgen and estrogen receptors to be a subset of the potential landscape of molecular targets in rodents. Steroid homeostasis and xenobiotic metabolism, developmental regulation, vascularization and angiogenesis, inflammation, and neural activity provide a more comprehensive set of predicted modes of action underlying the TDS hypothesis. Further multi-scale modeling of MRDT will be needed to capture the spatiotemporal dynamics of male reproductive tract development and disruption. In combination with further HTS experiments that cover more molecular targets in embryonic development, this approach provides novel mechanistic insights for male reproductive developmental disorders.

## Supplemental Material

(569 KB) PDF
