# Supplementary material for: Systems Toxicology of Male Reproductive Development: Profiling 774 Chemicals for Molecular Targets and Adverse Outcomes
Source: Environ Health Perspect. 2015 Dec 11;124(7):1050–61. doi: 10.1289/ehp.1510385 (PMC4937872; doi:10.1289/ehp.1510385)
Supplement: (569 KB) PDF [file ehp.1510385.s001.acco.zip › Supplemental Code and Data Index File.pdf]

Environ Health Perspect
DOI: 10.1289/ehp.1510385

Systems Toxicology of Male Reproductive Development: Profiling 774 Chemicals for Molecular Targets and Adverse Outcomes

Maxwell C.K. Leung, Jimmy Phuong, Nancy C. Baker, Nisha S. Sipes, Gary R. Klinefelter, Matthew T. Martin, Keith W. McLaurin, R. Woodrow Setzer, Sally Perreault Darney, Richard S. Judson, and Thomas B. Knudsen

Supplemental Code and Data Index

Excel File S1. PubMed articles of ToxRefDB chemicals by male reproductive endpoints.

Excel File S2. Malformation.
